# Serum Biomarker gMS-Classifier2: Predicting Conversion to Clinically Definite Multiple Sclerosis

**DOI:** 10.1371/journal.pone.0059953

**Published:** 2013-03-28

**Authors:** Georgina Arrambide, Carmen Espejo, Jennifer Yarden, Ella Fire, Larissa Spector, Nir Dotan, Avinoam Dukler, Alex Rovira, Xavier Montalban, Mar Tintore

**Affiliations:** 1 Department of Neurology-Neuroimmunology, Multiple Sclerosis Centre of Catalonia (Cemcat), Vall d’Hebron University Hospital and Research Institute, Universitat Autònoma de Barcelona, Barcelona, Spain; 2 Department of Research and Development, Glycominds, Modi’in, Israel; 3 Department of Research and Development, Glycominds, Simi Valley, California, United States of America; 4 Magnetic Resonance Unit (IDI), Vall d’Hebron University Hospital, Barcelona, Spain; Innsbruck Medical University, Austria

## Abstract

**Background:**

Anti-glycan antibodies can be found in autoimmune diseases. IgM against glycan P63 was identified in clinically isolated syndromes (CIS) and included in gMS-Classifier2, an algorithm designed with the aim of identifying patients at risk of a second demyelinating attack.

**Objective:**

To determine the value of gMS-Classifier2 as an early and independent predictor of conversion to clinically definite multiple sclerosis (CDMS).

**Methods:**

Data were prospectively acquired from a CIS cohort. gMS-Classifier2 was determined in patients first seen between 1995 and 2007 with ≥ two 200 µL serum aliquots (N = 249). The primary endpoint was time to conversion to CDMS at two years, the factor tested was gMS-Classifier2 status (positive/negative) or units; other exploratory time points were 5 years and total time of follow-up.

**Results:**

Seventy-five patients (30.1%) were gMS-Classifier2 positive. Conversion to CDMS occurred in 31/75 (41.3%) of positive and 45/174 (25.9%) of negative patients (p = 0.017) at two years. Median time to CDMS was 37.8 months (95% CI 10.4–65.3) for positive and 83.9 months (95% CI 57.5–110.5) for negative patients. gMS-Classifier2 status predicted conversion to CDMS within two years of follow-up (HR = 1.8, 95% CI 1.1–2.8; p = 0.014). gMS-Classifier2 units were also independent predictors when tested with either Barkhof criteria and OCB (HR = 1.2, CI 1.0–1.5, p = 0.020) or with T2 lesions and OCB (HR = 1.3, CI 1.1–1.5, p = 0.008). Similar results were obtained at 5 years of follow-up. Discrimination measures showed a significant change in the area under the curve (ΔAUC) when adding gMS-Classifier2 to a model with either Barkhof criteria (ΔAUC 0.0415, p = 0.012) or number of T2 lesions (ΔAUC 0.0467, p = 0.009), but not when OCB were added to these models.

**Conclusions:**

gMS-Classifier2 is an independent predictor of early conversion to CDMS and could be of clinical relevance, particularly in cases in which OCB are not available.

## Introduction

Evidence exists that both the number of lesions observed using baseline magnetic resonance imaging (MRI) [1], 2 and the presence of IgG oligoclonal bands (OCB) in the cerebrospinal fluid (CSF) [3]–[5] of patients with clinically isolated syndromes (CIS) are independent predictors of conversion to clinically definite multiple sclerosis (CDMS). However, MS is a highly heterogeneous disease, and the search for other biomarkers that could improve the prediction of conversion to CDMS may still be necessary for early and appropriate therapeutic decision making [6], [7].

A complex array of covalently attached glycans densely covers the surface of all cells and many proteins, and these molecules are a major component of the extracellular matrix. Thus, glycans are a prime antigen source and play a vital role in immunity. Indeed, antibodies against these molecules have been implicated in a number of autoimmune diseases [8], for example, those directed against galactose in collagen type II in rheumatoid arthritis [9], ganglioside GQ1b in Miller-Fisher syndrome [10] and oligomannose, mannobioside, laminaribioside, chitobioside, laminarin and chitin epitopes in Crohn’s disease [11], [12]. IgM antibodies directed against glycans composed of alpha-glucose disaccharides have been found in MS patients and demonstrated to distinguish relapsing-remitting MS patients from those with other neurological diseases [13]–[15]. One of the identified antibodies was directed against P63, a polymer of alpha-glucose molecules comprising two different carbohydrate structures [Glc(αl,6)Glc(α) and Glc(αl,3)Glc(α)]. Thus, a classification rule named gMS-Classifier2, which is based on the combination of polyclonal serum IgM antibody levels against P63 and age, was developed after an exploration analysis of clinical data and anti-glycan antibody levels in samples collected in the “Betaferon® in Newly Emerging multiple sclerosis For Initial Treatment” (BENEFIT) trial. In this study, this classification rule identified CIS patients at higher risk of converting to CDMS during the first two years of disease evolution [16]. To validate these preliminary results, herein we aimed to analyze the gMS-Classifier2 predictive value for early conversion of CIS patients to CDMS and to determine whether gMS-Classifier2 is an independent predictor of conversion to CDMS.

## Patients and Methods

### Ethics Statement

This study received approval from the Clinical Research Ethics Committee (CREC) of Vall d’Hebron University Hospital and Research Institute (Comitè Ètic d'Investigació Clínica –CEIC- de l’Hospital Universitari Vall d’Hebron-Institut de Recerca). Participants provided their written informed consent to participate in this study.

The present study is based on longitudinal clinical, CSF, serum and MRI data prospectively acquired from a cohort of CIS patients which started in 1995. Patients presenting for the first time with monophasic neurologic symptoms of the type seen in MS were recruited at the Vall d’Hebron University Hospital in Barcelona, Spain. Inclusion criteria were as follows: a CIS suggestive of central nervous system (CNS) demyelination involving the optic nerve, brainstem, spinal cord or other topography that were not attributable to other diseases; age <50 years; and onset of symptoms within three months of both clinical and MRI examinations. Patients were seen every three to six months and if relapses occurred. IgG OCB were examined using agarose isoelectric focusing combined with immunoblotting. The remaining biological samples were stored at −80°C until testing. Brain MRIs were performed after the first demyelinating event and repeated after twelve months and five years of follow-up. From 2001 onwards, baseline cranial MRIs were performed at three months after the first demyelinating event. Further clinical, CSF and MRI assessments have been detailed elsewhere [1].

Cases were selected from the CIS cohort based on the following eligibility criteria: consecutive patients older than 18 years of age seen between 1995 and 2007, with a minimum of two available 200 µL stored serum aliquots that had not undergone previous thawing.

A diagnosis of conversion to CDMS was made when new symptoms occurred after an interval of at least one month and only when other diagnoses had been excluded [17]. Time of follow-up was calculated based on the difference between the date of the baseline visit and the date of the last visit. De-identified and coded serum samples obtained at the time of enrolment in the department-wide sample repository were shipped to Glycominds, Ltd. (Modi’in, Israel) for analysis. Clinical data were not shared with collaborators at Glycominds until after the results of the serological analysis had been returned.

Serum samples were thawed according to the following protocol to prevent IgM precipitation: i) Samples were allowed to reach room temperature; ii) Samples were incubated at 37°C for 2 hours; iii) Samples were vortexed to homogeneity. IgM antibody measurement is stable if these conditions are met for no more than two freeze-thaw cycles.

Levels of anti-P63 IgM antibodies were measured in IgG-depleted serum samples by enzyme immunoassay (EIA) in duplicate. Briefly, microtiter 96-well plates with immobilized P63 were prepared as described elsewhere [18]. IgG was depleted from the samples using rheumatoid factor removal reagent (Chemicon, Australia, Cat. RFRR) according to the manufacturer’s instructions. Following IgG removal, serum samples (using a dilution of 1∶600 instead of the 1∶1200 originally described) were dispensed into microtiter wells in duplicate, incubated for 180 minutes at 4°C, and washed with wash buffer. Bound antibodies were labelled with horseradish peroxidase (HRP)-conjugated goat anti-human IgM antibody, washed, and 3, 3′, 5, 5′-tetramethylbenzidine was added for detection. After 30 minutes, the enzymatic reaction was stopped by adding 1% sulphuric acid solution to the wells, and the optical density (OD) was read at 450 nm using a Victor 1420 plate reader (Wallac, Turku, Finland). Each plate included a 5-point calibration curve. Anti-P63 serum levels were reported in arbitrary EIA units (EU). gMS-Classifier2 units were calculated according to the following algorithm: [1.171 − 0.082×age in years at the time of blood collection]+[0.015 × anti-P63 (EU)]. The gMS-Classifier2 was considered positive when the number of units was equal to or greater than 0.289 [16], [19].

### Statistical Analysis

Parametric and nonparametric comparative statistics were performed depending on the normality of the distributions of the continuous variables. Fisher’s exact test was performed to compare categorical variables. Kaplan-Meier analysis was used to estimate cumulative survival probabilities and to construct survival plots. To assess whether gMS-Classifier2 can independently predict time to CDMS, a multivariate analysis using Cox proportional hazard regression was performed for both gMS-Classifier2 status (positive or negative) and continuous values. Baseline MRI parameters such as number of Barkhof Criteria (BC), the number of T2 lesions (0, 1–9, >9 lesions) and OCB were considered as potentially relevant covariates. Age was already included in the gMS-Classifier2 algorithm as a covariate; the role of gender and CIS topography as possible covariates was also evaluated. Time to event analysis was performed primarily at two years; it was additionally assessed at five years and total time of follow-up to evaluate the length of time during which the biomarker could be useful. To assess the clinical utility of gMS-Classifier2, a Hosmer and Lemeshow goodness-of-fit test was performed as a calibration measure for two models: one with number of Barkhof criteria, OCB and gMS-Classifier2; and one for number of T2 lesions, OCB and gMS-Classifier2. As discrimination measures, two ROC curve analyses were made: one model using number of Barkhof criteria, OCB and gMS-Classifier2 continuous units and compared with a model without OCB. The second ROC curve analysis compared one model using number of T2 lesions, OCB and gMS-Classifier2 continuous units versus another in which OCB were excluded. Statistical tests were performed at the 0.05 level of significance using the Statistical Package for the Social Sciences (SPSS Inc., Chicago, IL, USA) version 17.0.

## Results

Between 1995 and 2007, 723 patients were included in the CIS cohort. gMS-Classifier2 units were determined in a subgroup of 249 (34%) patients that met the present study’s selection criteria. The screened cohort was similar to the non-screened cohort in age, follow-up time, and proportion of both positive OCB and baseline number of Barkhof criteria. There were differences in the proportion of females and the topography of disease presentation (Table 1). When comparing the demographic variables between patients with positive and negative gMS-Classifier2 status, including median time of follow-up, there was a difference in the proportion of females and in the distribution of CIS topography, but since the gMS-Classifier2 hazard ratio (HR) estimate was not substantially modified when gender or topography were included in the model, it was not considered necessary to adjust the results for these clinical variables (data not shown). There was also a difference in the median time of follow-up; however, as it was of approximately 5 months between groups, it was not considered relevant when the total follow-up time was of up to 14 years.

**Table 1:** Demographic, clinical and MRI characteristics of screened and non-screened patients: gMS-Classifier2 serum assay.

Group characteristics (1995–2007)	Screened CIS cohort (N = 249)	Non-screened CIS cohort (N = 474)	p-value
**Mean age in years ± SD**	31.6±7.9	31.6±7.9	0.455
**Females (%)**	187 (75.1)	315 (66.5)	0.017
**Median follow-up in months (range)**	68.7 (0.53–177.0)	63.2 (0.30–171.2)	0.002
**Topography N (%):**			
ON	106 (42.6)	154 (32.5)	
Brainstem	51 (20.5)	144 (30.4)	
Spinal cord	65 (26.1)	122 (25.7)	
Other	27 (10.8)	54 (11.4)	0.014
Positive OCB N (%)*	152 (64.4)	181 (60.1)	0.311
Barkhof criteria on baseline MRI N (%):**			
0	88 (35.5)	173 (38.6)	
1–2	56 (22.6)	104 (23.2)	
3–4	104 (41.9)	171 (38.2)	0.601

Abbreviations: CIS = clinically isolated syndrome; SD = standard deviation; ON = optic neuritis; OCB = oligoclonal bands; MRI = magnetic resonance imaging. *The total number of patients with available cerebrospinal fluid for OCB determination was 236 for the screened CIS cohort and 301 for the non-screened CIS cohort. Percentages in the table correspond to these figures. **The total number of patients with available baseline MRI for Barkhof criteria determination was 248 for the screened CIS cohort and 448 for the total CIS cohort. Percentages in the table correspond to these figures.

### gMS-Classifier2 Status at Specified Time Points and Conversion to CDMS

The median value of gMS-Classifier2 was −0.24 units (range −2.3 to 5.5 units) and seventy-five patients (30.1%) were positive for gMS-Classifier2.

**Figure 1:**
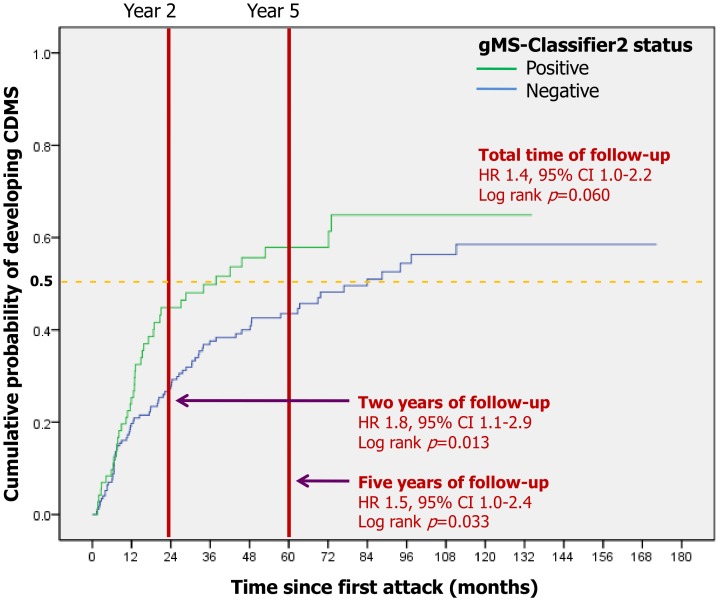
Time to reach CDMS based on gMS-Classifier2 status. Dotted line: median time of follow-up. HR = hazard ratio; CI = confidence interval.

The median time to CDMS was 37.8 months for gMS-Classifier2-positive patients (95%CI 10.4–65.3 months) and 83.9 months (95%CI 57.5–110.5) for gMS-Classifier2-negative patients. gMS-Classifier2 predicted conversion to CDMS within two years (HR = 1.8, 95%CI 1.1–2.9; p = 0.013) and within five years of follow-up (HR = 1.5, 95%CI 1.0–2.4; p = 0.033) but not for total follow-up time, although a trend was observed (HR = 1.4, 95%CI 1.0–2.2; p = 0.060) (Figure 1). Table 2 shows the proportion of CDMS patients that were positive and negative for gMS-Classifier2 at 2 years, 5 years and total time of follow-up.

**Table 2:** gMS-Classifier2 status and number of patients converting to CDMS at specified time points.

Specified time points	CDMS in positive patients (75)	CDMS in negative patients (174)	p*-*value
	N (%)	N (%)	
Two years	31 (41.3)	45 (25.9)	0.017
Five years	38 (50.7)	67 (38.5)	0.093
Total time of follow-up (up to 14 years)	40 (53.3)	77 (44.3)	0.214

Abbreviations: CDMS = clinically definite multiple sclerosis.

### Predictive Value of gMS-Classifier2 Status for Conversion to CDMS

In a univariate analysis, gMS-Classifier2 status (positive/negative), Barkhof criteria, number of T2 lesions and presence of OCB were predictors for early conversion to CDMS at two years of follow-up (Table 3).

**Table 3:** Univariate Cox proportional hazard regression for conversion to CDMS.

Univariate model	N	Two years of follow-up			Five years of follow-up			Total time of follow-up		
		HR	95%CI	*p*	HR	95%CI	*p*	HR	95%CI	*p*
gMS-Classifier2 status (positive or negative)	249	1.8	1.1–2.8	0.017	1.5	1.0–2.3	0.034	1.4	1.0–2.1	0.061
gMS-Classifier2 continuous units*	249	1.2	1.0–1.4	0.027	1.1	1.0–1.3	0.038	1.1	1.0–1.3	0.117
1–2 Barkhof criteria‡	56	3.9	1.6–9.4	0.002	4.3	2.1–8.6	<0.0001	5.3	2.7–10.6	<0.0001
3–4 Barkhof criteria	104	7.5	3.4–16.5	<0.0001	6.7	3.6–12.8	<0.0001	8.1	4.3–15.4	<0.0001
1–9 T2 lesions	76	5.6	1.7–18.9	0.005	10.0	3.1–32.6	<0.0001	11.5	3.6–37.4	<0.0001
≥10 T2 lesions	107	12.5	3.9–39.9	<0.0001	17.4	5.5–55.2	<0.0001	21.4	6.7–68.1	<0.0001
Positive OCB	152	3.7	1.9–7.3	<0.0001	3.1	1.8–5.3	<0.0001	2.9	1.8–4.8	<0.0001

Abbreviations: HR = hazard ratio, CI = confidence interval, OCB = oligoclonal bands.

* For continuous values, HR indicates how much the hazard (for CDMS) increases per unit increase in gMS-Classifier2.

‡ Barkhof criteria and number of T2 lesions on baseline MRI.

In the multivariate analyses at two years of follow-up, gMS-Classifier2 status remained significant when tested with number of Barkhof criteria (HR = 1.8, 95%CI 1.1–2.8, p = 0.014) or number of T2 lesions (HR = 1.7, 95%CI 1.1–2.7, p = 0.020). When combining gMS-Classifier2 status and OCB, the formeŕs significance was lost (HR = 1.5, 95%CI 0.9–2.4, p = 0.095). When combining gMS-Classifier2 status with OCB and either Barkhof criteria or number of T2 lesions the HR were non-significant (HR 1.5, 95%CI 0.9–2.5, p = 0.081 and HR 1.5, 95%CI 0.9–2.4, p = 0.100, respectively) (Table 4). When adding treatment to these models, there were no statistically significant changes in the HR of gMS-Classifier2 (data not shown).

**Table 4:** Multivariate Cox proportional hazard regression for conversion to CDMS according to gMS-Classifier2 status (positive or negative).

Multivariate models	Two years of follow-up			Five years of follow-up			Total time of follow-up		
	HR	95%CI	*p*	HR	95%CI	*p*	HR	95%CI	*P*
gMS-Classifier2 status	1.8	1.1–2.8	0.014	1.6	1.1–2.4	0.019	1.5	1.0–2.2	0.031
Number of Barkhof criteria, N = 1–2vs. 0‡	4.1	1.7–9.8	0.002	4.4	2.2–8.9	<0.0001	5.5	2.8–10.9	<0.0001
Number of Barkhof criteria, N = 3–4 vs. 0	7.5	3.4–16.5	<0.0001	6.9	3.6–13.1	<0.0001	8.3	4.4–15.7	<0.0001
gMS-Classifier2 status	1.7	1.1–2.7	0.020	1.5	1.0–2.3	0.044	1.4	1.0–2.1	0.074
Number of T2 lesions, N = 1–9 vs. 0	5.4	1.6–18.0	0.007	9.6	3.0–31.3	<0.0001	11.2	3.4–36.4	<0.0001
Number of T2 lesions, N≥10 vs. 0	12.2	3.8–39.1	<0.0001	17.2	5.4–54.8	<0.0001	21.4	6.7–67.9	<0.0001
gMS-Classifier2 status	1.5	0.9–2.4	0.095	1.3	0.9–2.0	0.217	1.2	0.8–1.8	0.307
Positive OCB	3.6	1.8–7.0	<0.0001	3.1	1.8–5.2	<0.0001	2.9	1.7–4.6	<0.0001
gMS-Classifier2 status	1.5	0.9–2.5	0.081	1.4	0.9–2.1	0.126	1.3	0.9–2.0	0.151
1–2 Barkhof criteria	3.1	1.2–7.6	0.015	3.5	1.7–7.3	0.001	4.4	2.2–9.0	<0.0001
3–4 Barkhof criteria	5.2	2.3–11.9	<0.0001	5.3	2.7–10.2	<0.0001	6.5	3.3–12.5	<0.0001
Positive OCB	2.2	1.1–4.5	0.022	1.9	1.1–3.4	0.014	1.8	1.1–2.9	0.023
gMS-Classifier2 status	1.5	0.9–2.4	0.100	1.3	0.9–2.0	0.204	1.2	0.8–1.9	0.267
Number of T2 lesions, n = 1–9 vs. 0	3.9	1.1–13.7	0.030	7.6	2.3–25.2	0.001	9.1	2.7–30.0	<0.0001
Number of T2 lesions, n≥10 vs. 0	8.2	2.5–27.1	0.001	13.1	4.0–42.7	<0.0001	16.5	5.1–53.3	<0.0001
Positive OCB	2.2	1.1–4.4	0.024	1.9	1.1–3.2	0.021	1.7	1.0–2.8	0.030

Abbreviations: HR = hazard ratio, CI = confidence interval, OCB = oligoclonal bands.

‡ Barkhof criteria and number of T2 lesions on baseline MRI.

### Predictive Value of gMS-Classifier2 Continuous Unit Values for Conversion to CDMS

In the univariate analysis, gMS-Classifier2 continuous units, Barkhof criteria, number of T2 lesions and presence of OCB were predictors for early conversion to CDMS at two and five years of follow-up (Table 3).

In the multivariate analyses at two years of follow-up, gMS-Classifier2 continuous units remained significant when tested with number of Barkhof criteria (HR = 1.3, 95%CI 1.1–1.5, p = 0.003) or number of T2 lesions (HR = 1.3, 95%CI 1.1–1.6, p = 0.001). When combining gMS-Classifier2 continuous units and OCB, the formeŕs significance was lost (HR = 1.1, 95%CI 0.9–1.4, p = 0.088), but when combining gMS-Classifier2 continuous units with OCB and either Barkhof criteria or number of T2 lesions the HR were once again statistically significant (HR 1.2, 95%CI 1.0–1.5, p = 0.020 and HR 1.3, 95%CI 1.1–1.5, p = 0.008, respectively) (Table 5).When adding treatment to these models, there were no statistically significant changes in the HR of gMS-Classifier2 (data not shown).

**Table 5:** Multivariate Cox proportional hazard regression models for CDMS conversion according to gMS-Classifier2 continuous units.

Multivariate models	Two years of follow-up			Five years of follow-up			Total time of follow-up		
	HR‡‡	95%CI	*p*	HR	95%CI	*p*	HR	95%CI	*p*
gMS-Classifier2 units‡	1.3	1.1–1.5	0.003	1.3	1.1–1.5	0.001	1.2	1.1–1.4	0.004
1–2 Barkhof criteria	4.5	1.8–10.9	0.001	4.9	2.4–10.0	<0.0001	6.0	3.0–12.0	<0.0001
3–4 Barkhof criteria	8.3	3.7–18.4	<0.0001	7.6	4.0–14.5	<0.0001	9.1	4.8–17.4	<0.0001
gMS-Classifier2 units	1.3	1.1–1.6	0.001	1.3	1.1–1.5	0.001	1.3	1.1–1.5	0.001
Number of T2 lesions, n = 1–9 vs. 0	6.0	1.8–20.5	0.004	10.7	3.3–34.8	<0.0001	12.4	3.8–40.3	<0.0001
Number of T2 lesions, n≥10 vs. 0	15.0	4.6–48.8	<0.0001	20.9	6.5–67.3	<0.0001	25.8	8.0–82.9	<0.0001
gMS-Classifier2 units	1.1	0.9–1.4	0.088	1.1	0.9–1.3	0.157	1.1	0.9–1.2	0.283
Positive OCB	3.4	1.9–7.2	<0.0001	3.1	1.8–5.2	<0.0001	2.9	1.8–4.7	<0.0001
gMS-Classifier2 units	1.2	1.0–1.5	0.020	1.2	1.0–1.4	0.015	1.2	1.0–1.4	0.020
1–2 Barkhof criteria	3.4	1.3–8.4	0.010	3.8	1.8–7.9	<0.0001	4.8	2.3–9.8	<0.0001
3–4 Barkhof criteria	5.7	2.5–13.1	<0.0001	5.8	2.9–11.3	<0.0001	7.1	3.6–13.8	<0.0001
Positive OCB	2.2	1.1–4.4	0.025	1.9	1.1–3.3	0.019	1.7	1.1–2.9	0.031
gMS-Classifier2 units	1.3	1.1–1.5	0.008	1.2	1.1–1.5	0.007	1.2	1.0–1.4	0.009
Number of T2 lesions, n = 1–9 vs. 0	4.4	1.3–15.2	0.020	8.3	2.5–27.6	0.001	9.9	3.0–32.9	<0.0001
Number of T2 lesions, n≥10 vs. 0	9.9	2.9–33.1	<0.0001	15.6	4.8–51.3	<0.0001	19.6	6.0–64.2	<0.0001
Positive OCB	2.2	1.1–4.3	0.029	1.8	1.1–3.1	0.031	1.7	1.0–2.7	0.044

Abbreviations: HR = hazard ratio, CI = confidence interval, OCB = oligoclonal bands.

‡ Barkhof criteria and number of T2 lesions on baseline MRI.

‡‡ For continuous values, HR indicates how much the hazard (for CDMS) increases per unit increase in gMS-Classifier2.

### Predictive Value of gMS-Classifier2 Status and Continuous Unit Values for Conversion to CDMS at Five Years and Total Time of Follow-up

Similar results were obtained at five years of follow-up in the uni- and multivariate analyses for gMS-Classifier2 status and continuous units (Tables 3, 4 and 5). At total time of follow-up, gMS-Classifier2 status and continuous units were not predictive of conversion to CDMS in the univariate analysis (Table 3), but when included in the multivariate models, gMS-Classifier2 continuous units remained independent predictors except when combined with OCB, whereas gMS-Classifier2 status yielded mostly negative results (Tables 4 and 5).

### Discrimination Measures: ROC Curve Analyses

To assess the clinical utility of gMS-Classifier2, the calibration measures for number of Barkhof criteria, OCB and gMS-Classifier2 continuous units yielded a p value of 0.303; and the one performed for number of T2 lesions, OCB and gMS-Classifier2 continuous units showed a p value of 0.664. When performing the ROC analyses as discrimination measures, in the model using number of Barkhof criteria, the ROC association statistics showed that when number of Barkhof criteria, OCB and gMS-Classifier2 were put together, the area under the curve (AUC) was 0.7786 (95%CI 0.7169–0.8403), in comparison with an AUC ROC of 0.7552 (95%CI 0.6945–0.8160) when not including gMS-Classifier2. Thus, the AUC change (ΔAUC) ROC was 0.0233 (p = 0.0788). But when the model excluded OCB, the results were the following: AUC ROC 0.7651 (95%CI 0.6990–0.8312) with gMS-Classifier2 versus AUC ROC 0.7236 (95%CI 0.6616–0.7856) without it, with a ΔAUC ROC of 0.0415, p = 0.012. Similar findings were observed when using number of T2 lesions instead of number of Barkhof criteria: when adding OCB to the model, the AUCs were 0.7855 (95%CI 0.7255–0.8455) with gMS-Classifier2 and 0.7528 (95%CI 0.6927–0.8128) without it, leading to a ΔAUC of 0.0328, p = 0.0515. When OCB were excluded from the model, the AUCs were 0.7733 (95%CI 0.7102–0.8364) and 0.7266 (95%CI 0.6669–0.7864), respectively, with a ΔAUC of 0.0467, p = 0.009.

## Discussion

Early administration of disease modifying therapies is highly recommended in CIS patients at risk for developing a second relapse [20], [21]. Therefore, although MRI remains the most important surrogate marker for predicting the risk of a second relapse in CIS patients [1], [2], the clinical outcome remains unpredictable due to the high variability of this disease among individuals. Thus, a need remains for auxiliary biomarkers that could provide additional information about the disease course [22], [23]. The presence of IgG OCB at baseline doubles the risk of developing a second attack independent of MRI, but in the revised 2010 diagnostic criteria for MS, CSF was only included in the diagnostic criteria for primary progressive MS. Thus, the testing of CSF may further decline, despite the fact that the International Panel agrees that the inclusion of CSF in the criteria requires further evaluation [5], [23]. Furthermore, an ideal biomarker should be non-invasive and simple to use, making potential serum prognostic markers a good option, as they would be easy to obtain [24].

Glycans are potential antigens, and indeed, antibodies against various types of glycans have been found in serum. Such antibodies were first described for the human blood group ABO antigens [25], and later findings have linked antibodies directed against glycans to several autoimmune diseases, either by association only or as etiopathogenic [8]–[12]. Consequently, diverse assays have been designed to identify anti-glycan antibodies in autoimmune diseases [18], [26] including MS [27]–[29]. Because some of these glycans are found within the type IV collagen matrix of the blood-brain barrier [14], it has been hypothesized that in MS patients, an inflammatory response could lead to the release of these carbohydrate antigens with the subsequent development of a humoral response [30]. Therefore, an array of glycans was screened in RRMS patients and healthy controls, observing that IgM antibodies to various alpha-glucose are elevated in the former group. Among the alpha-glucose, GAGA4 was the most notable and gMS-Classifier Dx was developed as GAGA4 normalized to total IgM. It was further analysed with other neurological diseases controls (13, 31), concluding that gMS-Classifier Dx differentiates between MS and non-MS patients. Based on previous experience in Crohn’s disease in which broader glycan structures increase performance and address different clinical utilities, gMS-Classifier Dx was extended to include the following anti-alpha glucose antibodies: GAGA2, 3, 4 and 6, thus establishing gMS-Classifier1, which is based on disaccharides covalently bound via a long linker (anti-GAGA2, anti-GAGA3, anti-GAGA4, anti-GAGA6) and was established for the prediction of “early relapse” (within 24 months) (14). Then, in the BENEFIT study, gMS-Classifier1 was analysed on three pre-defined end-points: 1) Time to CDMS, 2) Time to McDonald, and 3) Time to confirmed EDSS. However, Classifier1 was only significant on Time to confirmed EDSS (16, 32). Thus, Classifier1 seems to be more of a prognostic MS biomarker for progression rather than for diagnosis. As part of the BENEFIT study, several additional alpha-glucose antibodies were analysed due to previously found data that suggested it could be beneficial to further explore them and see their potential value. Among those additional alpha-glucose antibodies were P63, [a polymer based on Glc(α1–3)Glc(α) and Glc(α 1–6)Glc(α)], alpha-ramose, alpha-N-acetyl glucose and P64 [(a polymer based on Glc(α 1–4)Glc(α) and Glc(α 1–6)Glc(α)]. For each one, time to CDMS with a minimal criterion of 30% sensitivity and 90% specificity at 24 months was analysed in the BENEFIT placebo sub-cohort and on the entire cohort. Only P63 normalized to age predicted time to CDMS and it was called gMS-Classifier2 (16).

A logistic regression model for prediction of early conversion to CDMS was used to develop the classifier; the input data included a number of clinical variables such as age since previous data have shown that IgM levels vary considerably throughout the years [33], [34], plus the raw levels of anti-glycan IgM against 8 different glycan antigens. After backward selection only anti-P63 IgM levels and age were found to be independent variables which entered the model. Since this classification rule identified CIS patients at higher risk of converting to CDMS during the first two years of disease evolution [16], the aims of the present study were to confirm those results and to determine whether gMS-Classifier2 is an independent predictor of conversion to CDMS.

Our results show that in this hospital cohort, gMS-Classifier2 is an independent predictor for conversion of CIS patients to CDMS that could become a useful prognostic tool when tested within the first two years of disease evolution, and thus could add information to baseline MRI findings, more specifically, in cases in which lumbar puncture or OCB determination cannot be performed. gMS-Classifier2 was positive in 30% of CIS patients at baseline, and the median time to CDMS was approximately twice as short for gMS-Classifier2-positive patients than for negative patients. The predictive performance of gMS-Classifier2 was better during the early years of the disease and decreased with long-term follow-up. When gMS-Classifier2 status was evaluated together with MRI variables in the multivariate analyses at two and five years of follow-up, it remained an independent predictor of conversion to CDMS, but not when evaluated with OCB. With MRI and OCB findings, continuous unit values of gMS-Classifier2 independently predicted the development of an early second relapse, indicating the increased risk of relapse with increased serum levels of the biomarker. When performing the ROC analyses, the model for gMS-Classifier units was statistically significant only when OCB were excluded. However, recent publications emphasize that testing for any improvement using discrimination measures such as the change in the area under the ROC curve is extremely conservative [36], [37]. Thus, we consider the HRs to be sufficient to support the role of gMS-Classifier2 as an independent predictor of conversion to CDMS.

As for the added value of gMS-Classifier2 to OCB findings in predicting early CDMS conversion, the differing results obtained are probably partly due to the higher resolution of a biomarker that is measured in continuous units compared to a dichotomous biomarker [35].

We conclude that gMS-Classifier2 is an independent predictor for conversion of CIS patients to CDMS in the first years of the disease course and therefore could be of clinical relevance to determine which patients are at higher risk, particularly in cases in which OCB are not available.

## References

[1] Tintoré M, Rovira A, Río J, Nos C, Grivé E, et al (2006) Baseline MRI predicts future attacks and disability in clinically isolated syndromes. Neurology 67: 968–972. 17000962 10.1212/01.wnl.0000237354.10144.ec

[2] Fisniku LK, Brex PA, Altmann DR, Miszkiel KA, Benton CE, et al (2008) Disability and T2 MRI lesions: a 20-year follow-up of patients with relapse onset of multiple sclerosis. Brain 131: 808–817. 18234696 10.1093/brain/awm329

[3] Sharief MK, Thompson EJ (1991) The predictive value of intrathecal immunoglobulin synthesis and magnetic resonance imaging in acute isolated syndromes for subsequent development of multiple sclerosis. Ann Neurol 29: 147–151. 2012384 10.1002/ana.410290206

[4] Frederiksen JL, Larsson HB, Olesen J (1992) Correlation of magnetic resonance imaging and CSF findings in patients with acute monosymptomatic optic neuritis. Acta Neurol Scand 86: 317–322. 1414254 10.1111/j.1600-0404.1992.tb05093.x

[5] Tintoré M, Rovira A, Río J, Tur C, Pelayo R, et al (2008) Do oligoclonal bands add information to MRI in first attacks of multiple sclerosis? Neurology 70: 1079–1083. 17881717 10.1212/01.wnl.0000280576.73609.c6

[6] Arnold AC (2005) Evolving management of optic neuritis and multiple sclerosis. Am J Ophthalmol 139: 1101–1108. 15953446 10.1016/j.ajo.2005.01.031

[7] Tumani H, Hartung HP, Hemmer B, Teunissen C, Deisenhammer F, et al (2009) Cerebrospinal fluid biomarkers in multiple sclerosis. Neurobiol Dis 35: 117–127. 19426803 10.1016/j.nbd.2009.04.010

[8] van Kooyk Y, Rabinovich GA (2008) Protein-glycan interactions in the control of innate and adaptive immune responses. Nat Immunol 9: 593–601. 18490910 10.1038/ni.f.203

[9] Dzhambazov B, Nandakumar KS, Kihlberg J, Fugger L, Holmdahl R, et al (2006) Therapeutic vaccination of active arthritis with a glycosylated collagen type II peptide in complex with MHC class II molecules. J Immunol 176: 1525–1533. 16424181 10.4049/jimmunol.176.3.1525

[10] Chiba A, Kusunoki S, Shimizu T, Kanazawa I (1992) Serum IgG antibody to ganglioside GQ1b is a possible marker of Miller Fisher syndrome. Ann Neurol 31: 677–679. 1514781 10.1002/ana.410310619

[11] Sendid B, Colombel JF, Jacquinot PM, Faille C, Fruit J, et al (1996) Specific antibody response to oligomannosidic epitopes in Crohn’s disease. Clin Diagn Lab Immunol 3: 219–226. 8991640 10.1128/cdli.3.2.219-226.1996 PMC170283

[12] Rieder F, Schleder S, Wolf A, Dirmeier A, Strauch U, et al (2010) Serum anti-glycan antibodies predict complicated Crohn’s disease behavior: a cohort study. Inflamm Bowel Dis 16: 1367–1735. 20024902 10.1002/ibd.21179

[13] Schwarz M, Spector L, Gortler M, Weisshaus O, Glass-Marmor L, et al (2006) Serum anti-Glc(alpha1,4)Glc(alpha) antibodies as a biomarker for relapsing-remitting multiple sclerosis. J Neurol Sci 244: 59–68. 16480743 10.1016/j.jns.2005.12.006

[14] Freedman MS, Laks J, Dotan N, Altstock R, Dukler A, et al (2009) Anti-alpha-glucose-based glycan IgM antibodies predict relapse activity in multiple sclerosis after the first neurological event. Mult Scler 15: 422–430. 19324980 10.1177/1352458508101944 PMC2850589

[15] Brettschneider J, Jaskowski TD, Tumani H, Abdul S, Husebye D, et al (2009) Serum anti-GAGA4 IgM antibodies differentiate relapsing remitting and secondary progressive multiple sclerosis from primary progressive multiple sclerosis and other neurological diseases. J Neuroimmunol 217: 95–101. 19879655 10.1016/j.jneuroim.2009.07.017

[16] Freedman M, Edan G, Hartung HP, Kappos L, Miller DH, et al (2009) Anti-alpha-glucose based glycan IgM antibodies in patients with a clinically isolated syndrome: analyses from the Betaferon® in Newly Emerging multiple sclerosis For Initial Treatment (BENEFIT) study. Mult Scler. 15 (suppl 2)S71.

[17] Poser CM, Paty DW, Scheinberg L, McDonald WI, Davis FA, et al (1983) New diagnostic criteria for multiple sclerosis: guidelines for research proposals. Ann Neurol 13: 227–231. 6847134 10.1002/ana.410130302

[18] Schwarz M, Spector L, Gargir A, Shtevi A, Gortler M, et al (2003) A new kind of carbohydrate array, its use for profiling antiglycan antibodies, and the discovery of a novel human cellulose-binding antibody. Glycobiology 13: 749–754. 12851287 10.1093/glycob/cwg091

[19] Yarden J, Dotan N, Fire E, L Spector, H Schmidt, et al (2010) Positive serum values of gMS-Classifier2 associates with high relapse activity in relapsing-remitting multiple sclerosis patients. Mult Scler 16 (suppl (10)) S187.

[20] Frohman EM, Havrdova E, Lublin F, Barkhof F, Achiron A, et al (2006) Most patients with multiple sclerosis or a clinically isolated demyelinating syndrome should be treated at the time of diagnosis. Arch Neurol 63: 614–619. 16606781 10.1001/archneur.63.4.614

[21] Río J, Comabella M, Montalban X (2011) Multiple sclerosis: current treatment algorithms. Curr Opin Neurol 24: 230–237. 21499098 10.1097/WCO.0b013e328346bf66

[22] Fazekas F, Baumhackl U, Berger T, Deisenhammer F, Fuchs S, et al (2010) Decision-making for and impact of early immunomodulatory treatment: the Austrian Clinically Isolated Syndrome Study (ACISS). Eur J Neurol 17: 852–860. 20100231 10.1111/j.1468-1331.2009.02943.x

[23] Polman CH, Reingold SC, Banwell B, Clanet M, Cohen JA, et al (2011) Diagnostic criteria for multiple sclerosis: 2010 revisions to the McDonald criteria. Ann Neurol 69: 292–302. 21387374 10.1002/ana.22366 PMC3084507

[24] Teunissen CE, Dijkstra C, Polman C (2005) Biological markers in CSF and blood for axonal degeneration in multiple sclerosis. Lancet Neurol 4: 32–41. 15620855 10.1016/S1474-4422(04)00964-0

[25] Pettenkofer HJ, Bickerich R (1960) On common antigen properties between the human blood group ABO and the pathogen of diseases dangerous to the community. Zentralbl Bakteriol 179: 433–436. 13854323

[26] Nimrichter L, Gargir A, Gortler M, Altstock RT, Shtevi A, et al (2004) Intact cell adhesion to glycan microarrays. Glycobiology 14: 197–203. 14638630 10.1093/glycob/cwh022

[27] Menge T, Lalive PH, von Büdingen HC, Cree B, Hauser SL, et al (2005) Antibody responses against galactocerebroside are potential stage-specific biomarkers in multiple sclerosis. J Allergy ClinImmunol 116: 453–459. 10.1016/j.jaci.2005.03.023 16083805

[28] Lolli F, Mulinacci B, Carotenuto A, Bonetti B, Sabatino G, et al (2005) An N-glucosylated peptide detecting disease-specific autoantibodies, biomarkers of multiple sclerosis. Proc Natl Acad Sci U S A 102: 10273–10278. 16014416 10.1073/pnas.0503178102 PMC1177382

[29] Lolli F, Mazzanti B, Pazzagli M, Peroni E, Alcaro MC, et al (2005) The glycopeptide CSF114(Glc) detects serum antibodies in multiple sclerosis. J Neuroimmunol 167: 131–137. 16051375 10.1016/j.jneuroim.2005.05.016

[30] van Horssen J, Bö L, Dijkstra CD, de Vries HE (2006) Extensive extracellular matrix depositions in active multiple sclerosis lesions. Neurobiol Dis 24: 484–491. 17005408 10.1016/j.nbd.2006.08.005

[31] Brettschneider J, Jaskowski TD, Tumani H, Abdul S, Husebye D, et al (2009) Serum anti-GAGA4 IgM antibodies differentiate relapsing remitting and secondary progressive multiple sclerosis from primary progressive multiple sclerosis and other neurological diseases. J Neuroimmunol 217: 95–101. 19879655 10.1016/j.jneuroim.2009.07.017

[32] Freedman M, Metzig C, Kappos L, Polman C, Edan G, et al (2012) Predictive nature of IgM anti-α-glucose serum biomarker for relapse activity and EDSS progression in CIS patients: a BENEFIT study analysis. Mult Scler 18: 966–973. 22183938 10.1177/1352458511432327 PMC3546632

[33] Ritchie RF, Palomaki GE, Neveux LM, Navolotskaia O, Ledue TB, et al (1998) Reference distributions for immunoglobulins A, G, and M: a practical, simple, and clinically relevant approach in a large cohort. J Clin Lab Anal 12: 363–370. 9850188 10.1002/(SICI)1098-2825(1998)12:6<363::AID-JCLA6>3.0.CO;2-X PMC6808031

[34] Ritchie RF, Palomaki GE, Neveux LM, Navolotskaia O (1998) Reference distributions for immunoglobulins A, G, and M: a comparison of a large cohort to the world’s literature. J Clin Lab Anal 12: 371–377. 9850189 10.1002/(SICI)1098-2825(1998)12:6<371::AID-JCLA7>3.0.CO;2-T PMC6807938

[35] Simel DL, Samsa GP, Matchar DB (1993) Likelihood ratios for continuous test results–making the clinicians’ job easier or harder? J Clin Epidemiol 46: 85–93. 8433118 10.1016/0895-4356(93)90012-p

[36] Vickers AJ, Cronin AM, Begg CB (2011) One statistical test is sufficient for assessing new predictive markers. BMC Med Res Methodol 11: 13. 21276237 10.1186/1471-2288-11-13 PMC3042425

[37] Pepe MS, Kerr KF, Longton G, Wang Z (2013) Testing for improvement in prediction model performance. Stat Med Jan 7 [Epub ahead of print]. 10.1002/sim.5727 PMC3625503 23296397

